# Supplementary material for: Rebuilding Gaza’s health system: A qualitative study of healthcare workers’ experiences and lessons learned from responding to mass casualty incidents (2018–2021)
Source: PLoS One. 2026 Feb 17;21(2):e0342654. doi: 10.1371/journal.pone.0342654 (PMC12912611; doi:10.1371/journal.pone.0342654)
Supplement: Attachment — Submitted filename: Reviewer comments.pdf [file pone.0342654.s002.pdf]

Rebuilding Gaza's health system: a qualitative study of healthcare workers' experiences and lessons learned from responding to mass casualty incidents (2018–2021)

Reviewer comments:

Dear Authors,

I enjoyed reading your paper titled "Rebuilding Gaza's health system: a qualitative study of healthcare workers' experiences and lessons learned from responding to mass casualty incidents (2018–2021)". Your paper contributes to the literature by providing empirical evidence and insights from those with direct experience of managing the health system during and after repeated MCIs and other health emergencies in Gaza. The paper offers valuable guiding recommendations for current and future planning around the reconstruction of the health system in Gaza based on the experiences of HCWs. However, I have a few comments to help improve the paper.

Major Comments:

1. The manuscript uses WHO's building blocks plus a seventh "Gaza-specific" theme as the framework for analysis. However, the framework is introduced without specific justification. It is unclear how the Gaza-specific category interacts with the other building blocks. How did the Gaza-specific theme emerge inductively from the data?

2. The sample size needs more discussions. Although participants were recruited using purposive and snowballing sampling, the manuscript does not explain whether this recruitment approach yields a sample that is representative of healthcare workers operating in the specific context of Gaza.

3. As mentioned in the previous point regarding sample representativeness, the authors note the gender imbalance in their sample in the Discussion section. However, they do not provide any indication of the extent of gender imbalance among HCWs in Gaza overall, making it difficult to assess whether the sample reflects the underlying population structure.

Minor Comments:

1. Define "building blocks" the first time it appears in the Data Analysis subsection.

2. The figure’s readability could be improved. The authors may consider using white background blocks with black text to enhance contrast and make the information easier to read.
